# Effect of rapamycin on aging and age-related diseases—past and future

**DOI:** 10.1007/s11357-020-00274-1

**Published:** 2020-10-10

**Authors:** Ramasamy Selvarani, Sabira Mohammed, Arlan Richardson

**Affiliations:** 1 Department of Biochemistry & Molecular Biology, University of Oklahoma Health Sciences Center, Oklahoma City, OK USA; 2 Oklahoma City VA Medical Center, Oklahoma City, OK USA

**Keywords:** Rapamycin, Aging, Neurodegeneration, Lifespan, Cancer, Heart

## Abstract

In 2009, rapamycin was reported to increase the lifespan of mice when implemented later in life. This observation resulted in a sea-change in how researchers viewed aging. This was the first evidence that a pharmacological agent could have an impact on aging when administered later in life, i.e., an intervention that did not have to be implemented early in life before the negative impact of aging. Over the past decade, there has been an explosion in the number of reports studying the effect of rapamycin on various diseases, physiological functions, and biochemical processes in mice. In this review, we focus on those areas in which there is strong evidence for rapamycin’s effect on aging and age-related diseases in mice, e.g., lifespan, cardiac disease/function, central nervous system, immune system, and cell senescence. We conclude that it is time that pre-clinical studies be focused on taking rapamycin to the clinic, e.g., as a potential treatment for Alzheimer’s disease.

## Introduction

Rapamycin (also known by the trade names of sirolimus or rapamune) is a macrocyclic lactone produced by *Streptomyces hygroscopicus*, which was isolated from soil samples collected from Easter Island by Georges Nogrady in the late 1960s [1]. Scientists at Ayerst Pharmaceuticals in Canada discovered that *Streptomyces hygroscopicus* produced a compound that would kill fungi, which they named rapamycin after the name of Easter Island, Rapa Nui. The initial interest in rapamycin focused on its antifungal properties. When it was found that rapamycin inhibited the growth of eukaryote cells, research on rapamycin turned to rapamycin’s immunosuppressive and anticancer properties. Rapamycin was approved by the FDA in 1994 to prevent organ rejection in liver transplant patients. In addition to being used as an antirejection drug, rapamycin or its rapalogs are being used today to prevent restenosis after coronary angioplasty, and they are being tested in many clinical trials as antitumor agents, e.g., FDA approved the use of rapamycin in treatment of pancreatic cancer patients in 2011.

Research in the late 1980s turned to identifying the mechanism by which rapamycin blocked the growth of eukaryote cells. Heitman et al. [2] discovered the protein, target of rapamycin (TOR), in yeast that was responsible for rapamycin’s ability to inhibit growth. Three groups in 1994 independently identified the mammalian counterpart, mTOR [3–5]. TOR, a serine/threonine kinase, was found to be a master-regulator in the response of eukaryote cells to nutrients, growth factors, and cellular energy status, and this is now known as the TOR pathway. Harrison et al. [6] in 2009 reported that rapamycin increased the lifespan of both male and female mice. This was a major discovery in aging because it was the first evidence that the lifespan of a mammal could be significantly increased by a pharmacological agent. The journal, *Science*, selected this study as one of the major scientific break-throughs in 2009 (*Science* 326, 1598–1607), the first discovery in aging to be selected by *Science* as a break-through. Over the past decade, there has been an explosion in the number of reports studying the effect of rapamycin on aging and age-related diseases, and there have been several reviews describing various aspects of rapamycin on aging [7–11]. In this article, we review the data collected over the past decade on the effect of rapamycin on lifespan and age-related diseases.

## Effect of rapamycin on the lifespan of mice

The first data suggesting that rapamycin might affect longevity came from studies with invertebrates. In 2003, Vellai et al. [12] showed that a TOR mutation increased the lifespan of *Caenorhabditis* *elegans*, and other groups showed that mutations in TOR increased the lifespan of yeast [13] and *Drosophila* [14]. Subsequently, it was found that rapamycin increased lifespan in yeast [15]. Based on these data, David Sharp (University of Texas Health Science Center at San Antonio) proposed that the NIA Intervention Testing Program tests the effect of feeding rapamycin to mice. The study was initiated in 2006, and in 2009, Harrison et al. [6] reported the first data showing that feeding rapamycin (14 ppm or ~ 2.24 mg/kg based on average food consumption of mice) increased the lifespan of both male and female mice. Not only was this the first report to show that lifespan could be increased pharmacologically in both male and female mice, but more importantly, the increase in lifespan was observed when rapamycin was given to mice late in life (19 months). Up to this time, it was generally believed that initiating an intervention late life would have minimal impact on longevity, i.e., the manipulation would need to be initiated early in life before major age-related decrements occurred and the intervention maintained the rest of life. Interestingly, the increase in lifespan by rapamycin was similar when implemented at 4 months [16], 9 months [17], or 19 months [6] of age. Since the initial report in 2009, there have been fourteen additional studies showing that rapamycin increased the lifespans of male and female mice. As shown in Table 1, the effect of rapamycin on lifespan is robust because it has been replicated in many different laboratories with different strains of laboratory mice, ranging from inbred strains (e.g., C57BL/6 and 129) to the UM-HET3 mice (a heterogeneous strain of mice generated by a 4-way cross) and with different rapamycin dosing regimens. Only the study by Bitto et al. [20] reported that rapamycin had no effect on lifespan. At a high dose (8 mg/kg/day) given i.p., rapamycin had no effect of lifespan of female mice; however, this dose and route of rapamycin increased the lifespan of male rats 61%. On the other hand, 126 ppm of rapamycin given in the diet increased the lifespan of both female and male mice 39% and 45%, respectively. To our knowledge, there currently is no report showing that rapamycin has a negative effect on the lifespan of normal, laboratory strains of mice.

**Table 1:** Effect of rapamycin on lifespan of various strains of mice

Mouse strain	Age started	Rapa dose	Increase in life span	Reference
UM-HET3	19 months	14 ppm*	9% M–14% F	[6]
C57BL/6Nia	22–24 months	4 mg/kg i.p.+	> 100% M	[138]
UM-HET3	9 months	14 ppm*	10% M–18% F	[17]
129/Sv	2 months	1.5 mg/kg s.c.^	10% F	[40]
C57BL/6	4, 13, and 20 months	14 ppm*	11% M	[79]
UM-HET3	9 months	4.7, 14, and 42 ppm*	3–23% M to 16–26% F	[19]
C57BL/6J	4 months	14 ppm*	11% M–16% F	[16]
C57BL/6Nia	19 months	14 ppm*	0% M–6% F	[139]
129/Sv × C57BL/6	2 months	14 ppm*	12% M–21% F	[38]
C57BL/6Nia	20 months	126 ppm* 8 mg/kg i.p. daily^#^	45% M–39% F 61% M–0% F	[20]
C57BL/6J	20 months	2 mg/kg i.p.^@^	13% F	[10]
UM-HET3	9 months	14 ppm* + metformin*	23% M–23% F	[140]
C57BL/6 × C3H	20–23 months	4 mg/kg i.p.^++^	10% M–22%F	[30]
UM-HET3	9 months	4.7, 14, and 42 ppm*	16, 21, 26% F	[131]
C57BL/6	3 months	42 ppm*	58% M and F	[29]

*Rapamycin given in the diet

^Rapamycin given for 2 weeks and then not given rapamycin for 2 weeks

^#^Rapamycin given for only 3 months

^@^Rapamycin given 5 days/week

^+^Rapamycin given every other day for 6 weeks

^++^Rapamycin given every other day

Three additional points of interest with respect to rapamycin’s longevity affect can be seen from Table 1. First, rapamycin is effective over a wide dose range. Even at high doses, it does not have a negative effect on lifespan. Second, rapamycin increases the lifespan of both male and female mice, which is unique because of all of the other anti-aging interventions identified by the NIA Intervention Testing Program are sex specific, i.e., they significantly increased lifespan in one sex but have little or no effect on the other sex [18]. However, as the data in Table 1 show, the increase in lifespan is greater in female mice than male mice in those studies that have compared the effect of rapamycin on lifespan in both males and females. However, this difference becomes minimal at high doses of rapamycin [19, 20]. Thus, it appears that female mice tend to be more sensitive to the life-extending actions of rapamycin. Third, the study by Bitto et al. [20] showed that only 3 months of a high dose of rapamycin (126 ppm) late in life was able to increase lifespan dramatically, again pointing to the late life benefits of rapamycin as well as showing that rapamycin need not be continuously administered to mice for a beneficial effect.

Table 2 lists the studies showing that rapamycin significantly increased the lifespan of genetic mouse models that mimic various diseases in humans. Most of these studies used mouse models of accelerated cancer, and as would be expected, these studies show an increase in lifespan as well as reduction in the progression of the neoplastic tumors specific to each model. These studies are described in more detail below. In addition, five studies have used mouse models of other disease phenotypes. Using a mouse model (*Lmna*^*−/−*^) that mimics Hutchinson-Gilford progeria, Ramos et al. [21] showed that rapamycin increased lifespan over 50% and improved cardiac and skeletal muscle function in the *Lmna*^*−/−*^ mice. Khapre et al. [22] studied the effect of rapamycin on the lifespan of *Bmal1*^*−/−*^ mice because knocking out *Bmal1* (a transcription factor that is key to the circadian clock) increased mTORC1 activity and reduced lifespan and disrupted circadian rhythm. They showed that rapamycin increased the lifespan of the *Bmal1*^*−/−*^ mice leading Khapre et al. [22] to propose that the regulation of mTORC1 activity by Bmal1 is key to the circadian clock. Two groups have studied the effect of rapamycin on mouse models with mutations leading to mitochondrial dysfunction. Siegmund et al. [23] studied TK2^KI/KI^ mice, which have a nuclear mutation in the mitochondrial nucleotide salvage enzyme thymidine kinase resulting in reduced replication of mtDNA and mtDNA instability. Rapamycin dramatically increased the lifespan of the TK2^KI/KI^ without having any detectable improvement in mitochondrial dysfunction. The authors concluded that rapamycin enhanced longevity in the TK2^KI/KI^ mice through alternative energy reserves and/or triggering indirect signaling events. Johnson et al. [7] initially showed that rapamycin attenuated mitochondrial disease symptoms and progression in *Ndufs4*^*−/−*^ mice, which lack a subunit in mitochondria complex I and is a mouse model of Leigh syndrome. Johnson et al. [24] subsequently showed that rapamycin increased the lifespan of the *Ndufs4*^*−/−*^ mice, especially at very high doses of rapamycin, which were 28-fold higher than the dose of rapamycin initially showed to increase the lifespan of mice by Harrison et al. [6]. Reifsnyder et al. [25] studied a mouse model of type 2 diabetes, the BKS-*Lepr*^*db*^ mouse. They found that rapamycin doubled the lifespan of female mice but had no effect of the lifespan of male mice. Rapamycin improved both kidney and cardiac functions in the female BKS-*Lepr*^*db*^ mice.

**Table 2:** Effect of rapamycin on lifespan of various mouse models of human disease

Mouse strain	Age started	Rapa dose*	Increase in lifespan	Reference
Mouse models of cancer				
*Ptet*^*−/−*^	1 month	10 mg/kg/day by gavage	300% M and F	[42]
*Apc*^*D716*^	6–14 weeks	3 and 10 mg/kg/day^	140–220% M and F	[33]
*Apc*^*Min/+*^	Not given	40 mg/kg	78% M and F	[34]
HER-2/neu	2 months	1.5 mg/kg s.c.^#^	13% F	[40]
*p53*^*−/−*^	2 months	0.5 mg/kg by gavage^@^	30% M	[39]
*p53*^*+/−*^	< 5 months	1.5 mg/kg in water	28% M	[37]
*Rb1*^*+/−*^	8–10 weeks	14 ppm	14% M–9% F	[43]
*Apc*^*Min/+*^	50 days	14 and 42 ppm	280–440% F	[35]
HER-2/neu	4–5 months	045 mg/kg s.c.^#^	5–7% M and F	[41]
*p53*^*+/−*^	2 months	14 ppm	15% M – 17% F	[38]
*Rag2*^*−/−*^	3 months	14 ppm	120% M and F	[44]
*IFN-γ*^*−/−*^	5 months	14 ppm	34% M and F	[44]
Mouse models of diseases other than cancer				
*Lmna*^*−/−*^	2 months	14 ppm and 8 mg/kg i.p. every other day	23 and 57% M and F	[21]
*Bmal1*^*−/−*^	16 weeks	0.5 mg/kg in water	50% M and F	[22]
*Ndufs4*^*−/−*^	20 days	14 and 378 ppm	29 and 100% M and F	[24]
*TK2*^*KI/KI*^	15 days	4 mg/kg in water^+^	60% M and F	[23]
BKS-*Lepr*^*db*^	15 weeks	14 ppm	0% M–100% F	[25]

*Given in diet unless otherwise indicated

^Everolimus given by gavage 5 times a week

^#^Given 3 times a week for a period of 2 weeks followed by 2-week intervals without rapamycin

^@^Given 5 days followed by 9-day interval without treatment

^+^Given to dams at 0.8 mg/kg before weaning

Although the overwhelming majority of studies on the effect of rapamycin on longevity in mice have shown a significant increase in lifespan, there are five studies that have reported either no effect or reduced lifespan when treated with rapamycin. Two studies using transgenic mouse models of amyotrophic lateral sclerosis (G93A and H46R/H48Q) reported no increase in lifespan when given rapamycin [26, 27]. Sataranatarajan et al. [28] reported that 14 ppm, a dose of rapamycin that increases the lifespan of C57BL/6 mice, reduced the lifespan of the obese and diabetic C57BL/KsJ*lepr*^*db/db*^ mice, 13% in males and 15% in females. The reduced lifespan of the *db/db* mice by rapamycin was associated with an increase in suppurative inflammation, which was the primary cause of death in the *db/db* mice. Ferrara-Romeo et al. [29] reported that 42 ppm rapamycin reduced the lifespan of telomerase-deficient mice (G2-*Terc*^*−/−*^) 16% compared with over a 50% increase in the lifespan of the G2-*Terc*^*+/+*^ mice. Fang et al. [30] found that rapamycin reduced the lifespan of growth hormone receptor knockout (GHR-KO) mice (15% for males and 5% for females) even though the same dose of rapamycin increased the lifespan of the wild-type, control mice. The reduced lifespan of the GHR-KO mice was associated with impaired glucose and lipid homeostasis and increased inflammation.

## Effect of rapamycin on cancer in mouse models

Rapamycin would be predicted to reduce the progression of cancer because it has been shown to inhibit cell growth and proliferation. In addition, mTOR is frequently hyperactivated in cancer, and mTORC1 has often been observed to be deregulated in a wide variety of human cancers [31]. Data generated in the 3 years after the discovery that rapamycin increased lifespan in 2009 showed that rapamycin and rapalogs (e.g., everolimus, temsirolimus, ridaforolimus) attenuated various cancers induced in mice. A few of these studies are summarized in Table 3 showing that mTOR inhibitors have an antineoplastic effect on a broad range of cancers. For example, Rivera et al. [32] studied the effect of ridaforolimus on the growth of various human tumor xenografts in mice. They showed that the administration of ridaforolimus inhibited the growth of prostate (PC-3), colon (HCT-116), breast (MCF7), lung (A549), and pancreas (PANC-1) cancer cells.

**Table 3:** Ability of mTOR inhibitors to reduce cancer

mTOR inhibitor	Type of cancer	Effect of rapamycin	Reference
Rapamycin	Urothelial carcinoma	55% ↓ in tumor volume 40% ↓ in cell proliferation	[141]
Rapamycin	Anal carcinoma	↓ outgrowth of primary carcinoma	[142]
Rapamycin	Skin carcinoma (UV)	↓ outgrowth of primary carcinoma	[143]
Rapamycin	Breast cancer	↓ in tumor volume	[144]
Rapamycin	Pancreatic cancer	25% ↓ incidence of cancer	[145]
Rapamycin	Breast carcinoma with bone metastasis	↓ osteoclast population and osteolysis	[146]
Everolimus	Bladder carcinoma	↓ tumor growth	[147]
Temsirolimus	Mesothelioma	↓ tumor growth	[148]
Temsirolimus	NSC-lung carcinoma	↓ proliferation of carcinoma	[149]
Ridaforolimus	Carcinomas (prostate, breast, pancreatic, colon)	↓ tumor growth	[32]

The data in Table 2 show the effect of rapamycin (or everolimus) on the survival of various mouse models with genetically engineered mutations in genes involved in cancer. Particularly striking are the three studies with *APC*^*Min/+*^ (*Apc*^*D716*^) mice, which are a model of human colorectal cancer. Most human colorectal cancers have somatic mutations in the adenomatous polyposis coli (APC) tumor suppressor gene, and *APC*^*Min/+*^ mice develop multiple intestinal neoplasia. The *APC*^*Min/+*^ mice are relatively short lived, living a maximum of ~ 200 days compared with 800 to 900 days for normal laboratory mice. Three groups [33–35] showed that treating *APC*^*Min/+*^ mice with rapamycin or everolimus reduced intestinal neoplasia (polyp number and size) in the *APC*^*Min/+*^ mice. In addition, these studies showed that rapamycin or everolimus dramatically increased the lifespan of the *APC*^*Min/+*^ mice. For example, Hasty et al. [35] found that a high level of rapamycin (42 ppm) resulted in a lifespan longer than that observed in normal laboratory mice, over a fourfold increase in lifespan of the *APC*^*Min/+*^ mice.

Three groups have studied the effect of rapamycin on mice with deletions in the p53 gene, a transcription factor with broad biological functions, including as a tumor suppressor in humans [36]. Komarova et al. [37] and Christy et al. [38] found that rapamycin treatment resulted in a modest, but significant increase in the lifespan of *p53*^*+/−*^ mice. Komarova et al. [37] reported that rapamycin reduced the incidence of tumors in the *p53*^*+/−*^ mice; however, Christy et al. [38] did not observe any significant changes in tumor incidence in *p53*^*+/−*^ mice treated with rapamycin. Comas et al. [39] reported that rapamycin increased the lifespan of *p53*^*−/−*^ mice; however, Christy et al. [38] did not observe a significant increase in the lifespan of *p53*^*−/−*^ mice.

Two reports have described the effect of rapamycin on transgenic mice overexpressing *Her-2/neu*. HER2 is a member of the human epidermal growth factor receptor family and amplification/overexpression of this oncogene has been shown to play a role in certain types of breast cancer. Rapamycin treatment resulted in a modest, but significant increase in the lifespan of *Her-2/neu* transgenic mice [40, 41] and dramatically delayed the incidence of tumors in the *Her-2/neu* transgenic mice. Hernando et al. [42] reported that everolimus dramatically increased the lifespan of *Ptet*^*−/−*^ mice, a model of leiomyosarcomas. The *Ptet*^*−/−*^ mice develop widespread smooth muscle cell hyperplasia and abdominal leiomyosarcomas, and everolimus significantly reduced the growth rate of these tumors. Livi et al. [43] studied the effect of rapamycin on *Rb1*^*+/−*^ mice. The retinoblastoma gene (*Rb1*) was the first tumor suppressor gene identified in humans and prevents excessive cell growth by inhibiting cell cycle progression. Rapamycin increased the lifespan of the *Rb1*^*+/−*^ mice and reduced the incidence of thyroid C cell carcinomas as well as delaying the appearance and reducing the size of pituitary tumors. Hurez et al. [44] studied the effect of rapamycin on immunocompromised, cancer prone *Rag2*^*−/−*^, and *IFN-γ*^*−/−*^ mice. Cancer immune surveillance is reduced in these two mouse models and rapamycin increased the lifespan of both *Rag2*^*−/−*^ *and IFN-γ*^*−/−*^ mice; however, no data were presented on the effect of rapamycin on the incidence of tumors in these mice.

## Effect of rapamycin on cardiac function and disease in mice

The first indication that rapamycin might be important for the heart was the discovery that coronary stents coated with rapamycin prevented restenosis and stent thrombosis compared with non-coated or other drug-eluting stents [45], which led to FDA approval in 2003. Two other mTOR inhibitors (everolimus and zotarolimus) are currently used to prevent restenosis and thrombosis in patients who require coronary stents [46].

The effect of rapamycin and rapalogs on the cardiovascular system initially was not clear, especially in humans. In clinical studies with transplant patients, rapalogs induced a negative plasma cardiovascular risk profile, e.g., an increase in LDL cholesterol and triglyceride concentrations in plasma [47]. Rapamycin also has been reported to have deleterious effects on endothelial function (ability of a blood vessel to constrict and dilate) in laboratory animals and in human coronary arteries from sirolimus-eluting stents [48, 49]. Rapamycin also has been reported to accelerate senescence of endothelial progenitor cells [50]; however, as described below, most of the recent studies indicate that rapamycin reduces cellular senescence. Overall, these early studies are in conflict with the large number of studies in mice listed in Table 4 that have studied the effect of rapamycin on atherosclerosis in mice.

**Table 4:** Effect of rapamycin on heart disease and function in rodents

Mouse model	Rapa treatment	Effect of rapa treatment	Reference
Atherosclerosis			
*ApoE*^*−/−*^ fed high-cholesterol diet	*Mice (8 weeks) fed 50 or 100 μg/kg/day rapa for 8 weeks	Plaques reduced 48% by 100 μg/kg rapa.	[52]
*LDLR*^*−/−*^ fed high-cholesterol diet	Male mice (4 weeks) fed 0.05 and 1.5 mg/kg/day everolimus for 20 weeks	Plaques reduced 44 and 85% by 0.05 or 1.5 mg/kg everolimus.	[150]
*LDLR*^*−/−*^ fed high-cholesterol diet	*Mice (8 weeks) fed 0.1, 0.3, and 1.5 mg/kg/day rapa for 16 weeks	Plaques reduced 20 to 70% with increasing dose of rapa.	[53]
*LDLR*^*−/−*^ fed high-fat and cholesterol diet	Male and female mice (~ 9 weeks) fed 2.24 mg/kg/day rapa for 30 weeks.	Plaques reduced ~ 20% in both male and female mice.	[51]
Cardiomyopathy and hypertrophy			
Aortic banding of FVB/N mice	Male mice (12 weeks) given 2 mg/kg rapa i.p. for 1 day	Cardiac hypertrophy suppressed 67% by rapa.	[151]
Aortic banding of FVB/N mice	Male mice (12 weeks) given 2 mg/kg/day rapa i.p. for 1 week	Cardiac hypertrophy suppressed and cardiac function improved.	[152]
Aortic banding of SD rats	Male rats (8 weeks) given 1.5 mg/kg i.p. rapa for 1 day	Cardiac hypertrophy suppressed by rapa.	[153]
Aortic banding of caAT mice	*Mice (8 weeks) given 120 mg/kg/day i.p. rapa for 4 weeks	Cardiac hypertrophy reduced 32% by rapa.	[154]
Aortic banding of FVB/N mice	Male mice (12 weeks) given 2 mg/kg/day rapa by gavage for 4 weeks	Cardiac hypertrophy reduced over 50% by rapa.	[155]
*Ptpn11Y279C/+* (LS/+) mice	*Mice (8 or 12 weeks) given 2 mg/kg/day rapa i.p. for 2 weeks	Pathological cardiac hypertrophy reversed.	[156]
*Lmna*^*−/−*^ mice	*Mice (4 weeks) given 8 mg/kg rapa i.p. every other day for 1 week	Heart function improved (increase in LVEDD and LVESD).	[21]
Isoproterenol-treated SD rats	Male rats given 1.2 mg/kg/day rapa i.p. for 1 week	Cardiac hypertrophy prevented by rapa.	[157]
BKS-*Lepr*^*db*^ diabetic mice	Female mice (11 weeks) fed rapa for 16 weeks	Reduced cardiomyopathy and fibrosis.	[25]
Myocardial infarction			
Wistar rats	Male rats (14 weeks) given 3 mg/kg/day Everolimus for 4 weeks	Infarct size reduced and LV function improved.	[56]
C57BL/6 mice	Male mice (8–12 weeks) given 2–10 mg/kg/day rapa i.p. for 2 weeks	Improved cardiac function and reduced hypertrophy and fibrosis.	[57]
Heart function and aging			
Old (24 months) C57BL/6J mice	Female mice fed 2.24 mg/kg/day of rapa for 12 weeks	Ventricular function improved and hypertrophy reversed.	[58]
Old (25 months) C57BL/6 mice	Female and male mice fed 2.24 mg/kg/day rapa for 10 weeks	Reversed cardiac hypertrophy and diastolic dysfunction.	[59]
Old (22–25 months) C57BL/6 mice	Female and male mice fed 2.24 mg/kg/day rapa for 8 weeks	Improved diastolic function persisted 8 weeks after treatment.	[113]

*The sex of the mice was not given

Table 4 lists the studies that have examined the effect of rapamycin (or everolimus) on various aspects of heart disease/function in mice. Four groups have studied the effect of rapamycin or everolimus on the occurrence of atherosclerotic lesions in the aortic arch of either *ApoE*^*−/−*^ or *LDLR*^*−/−*^ mice fed a high-fat diet to induce atherosclerotic plaque formation. All four studies showed that rapamycin reduced aortic atheromas, and Jahrling et al. [51] found that this was paralleled by an improvement in cerebral blood flow and vascular density in *LDLR*^*−/−*^ mice fed a high-fat diet. Because treating transplant patients with rapalogs has been shown to increase blood levels of cholesterol and triglycerides, the four groups also measured blood levels of cholesterol in their mouse models of atherosclerosis. Three found that rapamycin treatment had no effect on blood levels of cholesterol or triglycerides groups [51–53]. Mueller et al. [54] reported that the blood levels of LDL and VLDL cholesterol were slightly higher in the everolimus-treated mice but observed no change in triglycerides. It is interesting to note that Ross et al. [55] observed no effect of rapamycin (1.0 mg/kg/day) treatment on blood triglyceride levels in the non-human primate, marmoset.

A large number of studies have evaluated the effect of rapamycin on cardiomyopathy and hypertrophy induced by physical, pharmacological, or genetic engineering in mice and rats. All nine studies show that rapamycin prevents or attenuates cardiomyopathy or hypertrophy in both mice and rats. Two studies examined the effect everolimus in rats or rapamycin in mice on myocardial infraction [56, 57]. Rapamycin improved cardiac function, reduced infarct size in rats, and reduced hypertrophy and fibrosis in mice.

The three studies on the effect of heart function in old mice are the most relevant to this review. The studies by Simon Melov’s group at the Buck Institute [58] and Peter Rabinovitch’s group at the University of Washington [59] showed that rapamycin treatment for 2.5 to 3 months attenuated cardiac dysfunction and reduced cardiac hypertrophy seen with age. In other words, short-term rapamycin treatment was able to reverse cardiac dysfunction and hypertrophy that occurred in the old mice. The ability of rapamycin to improve cardiac function is not limited to mice. Urfer et al. [60] showed that giving rapamycin (0.1 mg/kg, 3 times/week) for 10 weeks to middle-aged companion dogs improved both systolic and diastolic cardiac function. Recently, Rabinovitch’s group showed that the improvement in diastolic function after 2 months of rapamycin treatment of old mice persisted for 2 months after rapamycin treatment was discontinued, demonstrating that rapamycin can have lasting effects on cardiac function even after it is discontinued [113].

## Effect of rapamycin on the central nervous system of mice

Perhaps the most unanticipated aspect of rapamycin’s biological effects, besides its anti-aging actions, is its impact on the central nervous system in mice. The limited number of early studies suggested that rapamycin might have negative effects on memory because of its effect on protein synthesis [61]. However, as shown in Table 5, the current data overwhelmingly show that rapamycin has a positive effect on a variety of functions and diseases of the central nervous system. Salvatore Oddo and Veronica Galvan at the University of Texas Health Science Center at San Antonio independently reported the seminal studies in this area in 2010 when they showed rapamycin prevented the loss of cognition in mouse models of Alzheimer’s disease (transgenic AD mouse models). Each laboratory treated a different transgenic AD mouse with the same level of rapamycin that Harrison et al. [6] showed increased lifespan to determine if the longevity effects of rapamycin extended to attenuating Alzheimer’s disease. They found that feeding rapamycin for 2 to 3 months completely blocked the loss of memory in these transgenic AD mice that occurred at ~ 6 months of age [62, 63]. Subsequently, Oddo’s laboratory showed that the life-long feeding of rapamycin blocked the loss in cognition in old 3xTg-AD mice, i.e., cognition of old 3xTg-AD mice was not significantly different from old wild-type mice. The initial studies by the laboratories of Oddo and Galvan also showed that rapamycin treatment reduced the accumulation of Aβ aggregates in the brains of their transgenic AD mice [62, 63], which was expected because inhibition of mTOR signaling had been shown to induce autophagy [64]. Oddo’s laboratory also showed that rapamycin prevented tau pathology (tau phosphorylation) in the 3xTg-AD mice [62, 65]. Rapamycin was also found to prevent tau pathology in tau-specific mouse models, which overexpress human mutant tau genes [66, 67]. Galvan’s laboratory also showed that rapamycin restored cerebral blood flow and vascular density [127] and prevented the breakdown of the blood brain barrier in hAPP (J20) mice [158]. Thus, rapamycin has a global impact on the central nervous system in maintaining cognition in transgenic AD mice. It reduces Aβ and tau pathology, increases cerebral blood flow and vascularization, preserves the brain blood barrier, and reduces neuroinflammation by attenuating microglia and astrocyte activation [9].

**Table 5:** Effect of rapamycin on the central nervous system of mice

Model	Rapa dose	Effect of rapa treatment	Reference
Alzheimer’s disease			
3xTg-AD	14 ppm	Cognition improved; Aβ and tau pathology ameliorated.	[62]
hAPP (J20)	14 ppm	Cognition improved and Aβ aggregates reduced.	[63]
3xTg-AD	14 ppm	Life-long rapa reduced Aβ and tau pathology, and improved cognition.	[65]
hAPP (J20)	14 ppm	Restored CBF and vascular density, reduced Aβ, and improved cognition.	[127]
P301S	15 mg/kg i.p.	Reduced cortical tau tangles, forebrain insoluble, tau and astrogliosis.	[66]
APP/PS1	20 mg/kg, i.p.*	Enhanced Aβ clearance by autophagy and improved cognition	[128]
Tau P301L	15 mg/kg i.p.	Reduced tau-induced neuronal loss, synaptotoxicity, and astrogliosis.	[67]
APOE4	14 ppm	Improved CBF and blood brain barrier integrity and learning deficits.	[69]
hAPP (J20)*	14 ppm	Prevented blood brain barrier breakdown.	[158]
Ts65Dn	1 μ/animal internasal	Improved cognition and reduced Aβ pathology.	[70]
Parkinson’s disease			
C57BL + MPTP	7.5 mg/kg i.p.	Reduced the loss of TH^+^ neurons.	[71]
α-Synuclein overexpression	14 ppm	Improved forepaw stepping, rotarod, and pole test performances.	[73]
C57BL/6 + MPTP	7.5 mg/kg i.p.	Reduction in inflammatory cytokines.	[159]
C57BL/6 + MPTP	3 mg/kg i.p.	TH^+^ neurons increased and improvement in behavioral measurements of gait.	[72]
C57BL/6J + 6-OHDA	50 mg/kg i.p.	Depression- and anxiety-like behavior eliminated.	[160]
Huntington’s disease			
HD-N171-N82Q	20 mg/kg i.p.	Enhances performance of mice on grip, rotarod, wire walking, and tremors tests.	[74]
Neurovascular diseases			
*LDLR*^*−/−*^-fed high-fat diet	14 ppm	CBF and brain vascular density improved.	[51]
*LDLR*^*−/−*^-fed high-fat diet	14 ppm	Prevented blood brain barrier breakdown.	[158]
Brain injury (traumatic brain injury and drug and surgical induced)			
Mice-THC	1 mg/kg i.p.	Abrogated the amnesic-like effects of delta9-tetrahydrocannabinol (THC).	[161]
C57BL/6-TBI	10 mg/kg i.p.	Reduced neural stem cell proliferation induced by controlled cortical impact.	[162]
C57BL/6-surgical	5 mg/kg i.p.	Cognition improved in postoperative cognitive dysfunction.	[163]
Kunming-sepsis	1, 5, 10 mg/kg i.p.	Rescued learning and memory deficits.	[164]
C57BL/6-isoflurane	1 mg/kg/day i.p.	Cognition improved in isoflurane-induced cognitive impairment.	[165]
Neurodevelopmental disorders (autism, epilepsy, seizures, etc.)			
*Tsc2*^*+/−*^	1 mg/kg i.p.	Rescues synaptic plasticity and behavioral deficits in this autism model.	[81]
*Tsc1* mutants	Not given	Prevented autistic-like behaviors.	[82]
*Tsc1*^*+/−*^	6 mg/kg i.p.	Reduction in anxiety and depression.	[83]
Tsc1fl/fl X GFP-Cre	6 mg/kg/day i.p.	Increased neuronal migration and spine density in an autism model.	[84]
*Disc1*^*−/−*^	20 mg/kg i.p.	Reversed cognitive and affective deficits in model of schizophrenia/depression.	[166]
Sprague Dawley rats	6 mg/kg i.p.	Improved spatial learning and memory in pilocarpine-induced epilepticus	[167]
Cognition and aging			
C57BL6/129svj	14 ppm	Cognition improved in old (18 months) mice	[78]
C57BL/6J	14 ppm	Cognition improved in 8- and 25-month-old mice	[77]
C57BL/6JRj F344BNF1 rats	14 ppm 42 ppm (5 months) and then 14 ppm (10 months)	Cognition improved in 11- and 20-month-old mice Improved cognition, neurovascular uncoupling, and cerebral perfusion in 35-month-old rats	[79] [80]

*Used the rapalog, temsirolimus

Rapamycin has also been shown to affect mouse models related to Alzheimer’s disease. The apolipoprotein Eε4 allele (APOE4) is the major genetic risk factor for Alzheimer’s disease in humans; individuals with one or two copies of this allele have a fourfold to eightfold increased risk in developing Alzheimer’s disease [68]. Using transgenic mice expressing the human APOE4 gene, Lin et al. [69] showed that rapamycin improved CBF, blood brain barrier integrity, and cognition deficits in these mice. More recently, Tramutola et al. [70] studied the effect of rapamycin on Down syndrome, a genetic disease of trisomy 21 in which individuals develop Alzheimer-like dementia. Using a mouse model of Down syndrome (Ts65Dn mice), they showed that internasal delivery of rapamycin improved cognition of the Ts65Dn mice and reduced aberrant amyloid precursor protein levels and tau pathology.

As shown in Table 5, rapamycin has also been shown to have an impact on two other types of neurodegeneration: Parkinson’s and Huntington’s disease. Several studies have reported that rapamycin prevents various aspects of Parkinson’s disease in different mouse models of Parkinson’s disease. For example, rapamycin prevented the loss of tyrosine hydroxylase (TH^+^) neurons in the substantia nigra pars compacta [71, 72] and improved various measures of muscle coordination [72, 73]. Ravikumar et al. [74] showed that rapamycin protected against Huntington’s disease. Using both a *Drosophila* (*yw;gmr-Q120* line) and a mouse model (HD-N171-N82Q) of Huntington’s disease, they showed that rapamycin attenuated the polyglutamine (polyQ) toxicity in *yw;gmr-Q120 Drosophila* and enhanced various tests of motor performance in HD-N171-N82Q mice. Berger et al. [75] and King et al. [76] showed that treating cells that expressed polyQ with rapamycin enhanced the clearance of polyQ aggregates. Berger et al. [75] found similar results with *Drosophila* expressing polyQ proteins and showed that rapamycin was protective against tau protein in *Drosophila*. Sarkar et al. (2008) showed that lithium and rapamycin exert an additive protective effect against neurodegeneration in a *Drosophila* model of Huntington’s disease.

In addition to its positive effect on neurodegeneration, rapamycin also has a neuroprotective effective effect on neurovascular disease, brain injury, and neurodevelopmental disorders (Table 5). Of particular interest to this review was the unexpected observation that rapamycin attenuated the age-related decline in cognition in normal mice. Again, the laboratories of Veronica Galvan and Salvatore Oddo were the first to independently and simultaneously report that old mice treated with rapamycin showed a significant improvement in cognition. Galvan’s laboratory studied the effect of feeding rapamycin (14 ppm) on the cognition of C57BL/6 mice at 8 and 25 months of age [77]. Eight-month-old male mice treated with rapamycin for 4 months showed a significant improvement in cognition measured by the Morris water maze. When 25-month-old mice (combined males and females) were fed rapamycin starting at 21 months of age, a significant improvement in cognition as measured by passive avoidance test (response to a mild foot shock) was observed in the rapamycin-treated mice. Oddo’s laboratory treated mice (sex not given) with 14 ppm rapamycin starting at 2 months of age, and cognition was measured by the Morris water maze at 18 months of age [78]. Rapamycin resulted in a 30–40% improvement in cognition. The improvement in cognition was correlated to reduced expression of the proinflammatory cytokine, IL-1β. In contrast, they observed no impact of rapamycin on cognition in 18-month-old mice when rapamycin treatment was started at 15 months of age; however, other groups have reported an improvement in cognition when mice are given rapamycin later in life. Neff et al. [79] replicated the effect of rapamycin on cognition in mice previously reported by Oddo and Galvan. Neff et al. [79] studied the effect of rapamycin (14 ppm) on a variety of measures of cognition (Morris water maze, passive avoidance, and novel object recognition) in male mice. Rapamycin had no effect on the novel object recognition test; however, 11- or 20-month mice treated with rapamycin for 6 weeks performed significantly better on the Morris water maze than mice fed the control diets, and mice (15, 24, and 33 months old) treated with rapamycin for 12 weeks performed significantly better on the passive avoidance test. More recently, Galvan’s group studied the effect of rapamycin on various parameters of brain function in old rats [80]. They found that treating rats with rapamycin (42 ppm for 5 months and 14 ppm for 10 months) starting at 19 months of age prevented deficits in learning and memory, prevented neurovascular uncoupling, and restored cerebral perfusion in 34-month-old rats. They argued that changes in the brain with normal aging and Alzheimer’s disease involve a vascular mechanism and that rapamycin improves vascular integrity and function in normal aging and in the pathogenesis of Alzheimer’s disease.

An interesting observation came from the study by Halloran et al. [77] when they observed that rapamycin has a significant effect on behavior of male C57BL/6 mice. Rapamycin (14 ppm) reduced anxiety-like behavior (thigmotaxis and elevated plus maze) and depressive-like behavior (floating and tail suspension test) in 4- and 12-month-old mice. As shown in Table 5, several studies report that rapamycin reduced behavioral deficits such as anxiety and depression in mouse models of autism [81–84]. However, Hadamitzky et al. [85, 86] reported that rapamycin (3 mg/kg, i.p.) induced anxiety-like behaviors (elevated plus maze, open field) when given to young rats (DA/HanRj).

## Effect of rapamycin on the immune response to infectious agents in mice

Because rapamycin was first developed as part of a cocktail to prevent rejection in transplant patients, it is generally assumed that rapamycin is an immunosuppressant. Therefore, when it was observed that rapamycin increased the longevity of mice, there were questions about the translatability of using rapamycin to delay aging in humans because of its potential negative effect on the immune system. However, it is now recognized that rapamycin is best identified as an immunomodulator rather than an immunosuppressant [44, 87]. Because of its use in transplant patients and its potential anti-aging role, there has been a large number of studies on the effect of rapamycin and mTOR signaling on various aspects of the immune system, and there have been several recent review articles in this area [88–91]. In this review, we will focus on those studies that have evaluated the effect of rapamycin treatment on the ability of an animal to respond to an antigen/vaccine or infectious agent.

Table 6 shows that the overwhelming majority of cases show that rapamycin provided protection from a variety of infectious agents starting with the first study by Weichhart et al. [92]. Jagannath et al. [93] and Araki et al. [94] were the first groups to report that rapamycin increased vaccine efficacy in mice, which protected the animals from subsequent infection. The ability of rapamycin to increase vaccine efficacy has been shown by many groups [93, 95]. Araki et al. [94] also showed that rapamycin enhanced vaccine response in Rhesus macaques to the modified Ankara virus vaccine. More recently, Mannick et al. [96] showed that the response to the influenza vaccination was improved in human subjects ≥ 65 years of age with 6 weeks of treatment with the rapalog, RAD001. Patients receiving rapamycin also had higher antibody titers to influenza virus vaccine a year later [97].

**Table 6:** Effect of rapamycin on the immune response to antigens/vaccines and infectious agents

Mouse	Rapa treatment	Effect of rapamycin	Reference
Improved immune response			
BALB/c F, 7–8 weeks	1.5 mg/kg, i.p.	Protected genetically susceptible mice against lethal *Listeria monocytogenes* infection.	[92]
C57BL/6 M and F, 4–8 weeks	Rapa-DC*	Increased vaccine efficacy against tuberculosis and mice immunized showed enhanced protection.	[93]
C57BL/6 M, 8–12 weeks	5 mg/kg, i.p.	Less sever lung injury after intratracheal administration of LPS or PAM.	[168]
C57BL/6J 12–16 weeks	75 and 600 μg/kg, i.p	Increased antigen-specific T cell response to lymphocytic choriomeningitis virus (LCMV) infection.	[94]
C57BL/6 M, 6–8 weeks	1.5 μg/da, i.p.	Increased antigen-specific response to bacterial infection but not to skin graft.	[99]
C57BL/6 M and F 22–24 months	14 ppm	Reduced lung damage and mortality after infection with *Streptococcus pneumonia*.	[110]
C57BL/6 F, 8–10 weeks	75 μg/kg, i.p.	Increased clearance of influenza virus and survival when administered during immunization.	[95]
C57BL/6 8–12 weeks	75 μg/kg, i.p.	Improved bacterial clearance in the spleen after Lm-gp33 challenge.	[98]
C57BL/6 8–12 weeks	75 μg/kg, i.p.	Higher bacterial burden in the liver and spleen arising from the bacterial pathogen Lm-OVA.	[98]
C57BL/6 F, 6–8 weeks	1 mg/kg, i.p.	Improved the clinical symptoms of autoimmune encephalomyelitis and reduced inflammatory cell proliferation in the central nervous system.	[169]
C57BL/6 F, 10 weeks	0.15 mg, i.p.	Reduced immunosuppression and secondary infection to *Candida* induced by inflammation and sepsis.	[170]
Cd1d^−/−^ F, 10 weeks	0.15 mg, i.p.	Reduced secondary infection to *Candida* induced by inflammation and sepsis.	[170]
No effect on immune response			
RAG2^−/−^ 3 months	14 ppm	No effect on the survival to bacteria, *Citrobacter rodentium*.	[44]
Reduced immune response			
C57BL/6 M, 6 weeks	1.5 mg/kg, i.p.	Increased lung injury after LPS intratracheal administration.	[171]
C57BL/6 8–12 weeks	75 μg/kg, i.p.	Higher bacterial burden in the liver and spleen arising from the bacterial pathogen Lm-OVA.	[98]
C57BL/6 8–12 weeks	75 μg/kg, i.p.	Higher viral titer in the brain after West Nile virus (WNV) infection.	[98]
C57BL/6 M, 16–18 months	14 ppm	Reduced survival to WNV meningoencephalitis but the reduction was not statistically significant.	[172]

*Immunized mice with dendritic cells treated with 1 mM rapamycin for 2 h

One of the limitations in the current studies evaluating the effect of rapamycin on infectious agents is that almost all of the studies have used young mice (under 3 months of age). Two groups studied the effect of a same dose and formulation of rapamycin (14 ppm) used by Harrison et al. [6] on resistance to infectious agents in old mice. Orihuela’s group studied the effect of the rapamycin on pneumococcal pneumonia induced by *Streptococcus pneumonia*. Old male C57BL/6 mice (24 months) were treated with rapamycin for 4 or 20 months. Both groups of mice showed improved survival to pneumococcal pneumonia and reduced lung pathology; however, the increased survival was not statistically significant for the mice given rapamycin for 20 months. On the other hand, Goldberg et al. [98] found that treating old (18 months) male C57BL/6 mice for 2 months with rapamycin reduced the survival to West Nile virus (WNV); however, this decrease was not significant. Interestingly, caloric restriction, a manipulation that has been shown to increase lifespan and delay aging animals ranging from invertebrates to non-human primates, showed a greater reduction in survival to WNV than rapamycin. Thus, it is quite possible that rapamycin will have different effects with different infectious agents. This is supported by a study of Ferrer et al. [99] who compared the effect of rapamycin on the antigen-specific T cell response with a bacterial infection versus a transplant. They found that treatment with rapamycin augmented the antigen-specific T cell response to the bacteria but failed to do so when the antigen was presented in the context of a transplant. They concluded that the environment in which an antigen is presented affects the influence of rapamycin on antigen-specific T cell expansion.

## Effect of rapamycin on cell senescence

In 1961, Leonard Hayflick described the phenomenon of cell senescence when he showed that human fibroblasts did not grow indefinitely in culture but underwent irreversible growth arrest [100]. It was later shown that the shortening of telomeres was responsible for cellular senescence observed in the fibroblast cultures by triggering a DNA damage response [101]. Subsequently, it was shown that senescence could be induced in a variety of cells, even post-mitotic cells by DNA-damaging agents and activation of oncogenes. A major break-through occurred when Judith Campisi discovered that senescent cells exhibited a senescent associated secretory phenotype (SASP) in which they secreted a variety of inflammatory cytokines, growth factors, and proteases [102]. Because the number of senescent cells increase with age and the SASPs produced by senescent cells could play a role in the age-related increase in chronic inflammation, cell senescence might be an important mechanism underlying aging [103]. Therefore, investigators began studying whether rapamycin had an effect on cell senescence when it was discovered that rapamycin increased lifespan. Although Imanishi et al. [50] initially reported that rapamycin accelerated senescence of endothelial progenitor cells, the eighteen studies published since 2009 show that cell senescence is attenuated by rapamycin.

The studies listed in Table 7 show that rapamycin was able to reduce or block senescence in a variety of cells from humans, mice, and rats. In addition, rapamycin has been shown to be effective in suppressing senescence induced by a variety of agents such as replicative senescence, DNA-damaging agents (e.g., UV and ionizing radiation, H_2_O_2_, bleomycin), and oncogene activation. Cao et al. [104] also studied the effect of rapamycin on senescence induced by the accumulation of an abnormal lamin A protein (progerin) in fibroblasts isolated from patients with Hutchinson-Gilford progeria. Rapamycin enhanced the degradation of progerin, abolished nuclear blebbing in the cells, and delayed the onset of cellular senescence.

**Table 7:** Effect of rapamycin on cell senescence

Source	Cell type	Effect of rapamycin treatment	Reference
K5rtTA/tet-Wnt1 mice (in vivo)	Epithelial	Reduced number of senescent cells induced in mice by genetically by Wnt induction.	[109]
Human (cell lines)	ARPE-19, HT-p21, and HT1080	Prevented senescence induced by H2O2, p21, and butyrate.	[173]
Rodent	Fibroblast	Modest decrease in senescence induced by butyrate but prevented loss of proliferation.	[173]
Human	Fibroblast (WI-38)	Reduced replicative senescence.	[174]
Human	Fibrosarcoma (HT1080-p-21-9)	Suppressed senescence induced by p21.	[175]
Human	Breast Cancer (MCF-7)	Prevented senescent morphology in nutlin-treated cells.	[175]
Human	Fibroblast (HGADFN167)	Delayed senescence caused by progerin in cells from Hutchinson-Gilford progeria patient.	[104]
Mouse in vivo	Lung	Reduced cellular senescence induced by bacterial infection.	[110]
Rat	Mesangial cells	Prevented senescence induced by high glucose.	[176]
Human	Oral epithelial keratinocytes	Protected primary cultures of cells from UV-induced cell senescence.	[177]
Mouse	Oral epithelial	Reduced the level of senescent epithelial cells induced by UV irradiation.	[177]
Mouse	Embryonic cells	Suppressed replicative senescence but blocked proliferation of cells.	[178]
Human	Fibroblast (WI-38)	Increased replicative life span.	[179]
Human	Endothelial cells (HPAEC)	Blocked radiation-induced senescence.	[180]
Human	Fibroblasts (HCA2)	Suppressed the secretion of SASPs induced by ionizing radiation.	[105]
Human	Fibroblasts (IMR90)	Prevented the SASP phenotype of oncogene-induced senescent cells but not growth arrest.	[106]
Mouse	Embryonic fibroblasts	Inhibited stress (H2O2)-induced senescence and delays replicative senescence.	[181]
Mice in vivo	Aorta	Reversed the effect of age on p19 that is associated with cellular senescence.	[111]
Human	Pulmonary cells (P-ECs)	Inhibited cell senescence and SASP in cells isolated from COPD patients.	[107]
Zmpste24^−/−^ mice in vivo	Muscle stem cells	Reduced level of senescent cells in a mouse model of progeria syndrome.	[182]
Mouse	Lung epithelial cells (MLE-12)	Suppressed senescence and SASP in cells treated with bleomycin.	[108]
Mice in vivo	Lung	Impaired the expression of senescent markers in the lung of mice treated with bleomycin.	[108]

In addition to suppressing markers of senescence, such as p16 and p21 expressions and SA-β-gal-positive cells, rapamycin reduced/prevented the SASP phenotype, i.e., the expression and secretion of proinflammatory cytokines by senescent cells. Two groups independently and simultaneously reported in 2015 that rapamycin reduced SASP produced by senescent human fibroblasts. Campisi’s group at the Buck Institute reported that rapamycin suppressed the secretion of proinflammatory cytokines produced by a variety of human cells isolated from different tissues (foreskin, fetal lung, adult prostate, and breast epithelial cells) [105]. Because SASP factors can promote cancer cell proliferation in culture, they studied the effect of media from senescent prostate cancer cells (PSC27) treated or not treated with rapamycin on the growth and migration of various cancer cell lines. They found that rapamycin reduced proliferation, migration, and invasion of cells. They also implanted PSC27 senescent cells with PC3 prostate cancer cells subcutaneously into SCID mice. Rapamycin treatment of the senescent cells before implantation resulted in a 50% decrease in tumor growth. Gil’s group at the Imperial College London studied the effect of rapamycin on oncogene-induced senescence in human fibroblasts [106]. Rapamycin reduced markers of senescence and SASPs secreted by the senescent cells. Subsequently, two other laboratories also reported that rapamycin reduced SASP in senescent cells. Houssaini et al. [107] showed that rapamycin reduced the secretion of proinflammatory cytokines from senescent pulmonary vascular endothelial cells from patients with chronic obstructive pulmonary disease. Chen et al. [108] studied senescence in idiopathic pulmonary fibrosis using lung epithelial cells treated with bleomycin. Rapamycin suppressed markers of senescence in the bleomycin-treated cells and the expression of proinflammatory cytokines. In a co-culture system with the bleomycin-treated cells and pulmonary fibroblasts, rapamycin treatment attenuated the proliferation of pulmonary fibroblasts and decreased the expression of α-smooth muscle actin and collagen I in the fibroblasts compared with pulmonary fibroblasts co-cultured with bleomycin-treated cells not treated with rapamycin. Thus, the current data support rapamycin’s ability to reduce SASP expression by senescent cells.

Of particular interest to this review are the three studies showing that rapamycin suppressed cell senescence in vivo in mice. Castilho et al. [109] studied a genetically engineered mouse (K5rtTA/tet-Wnt) in which Wnt1 is persistently expressed in the epithelial compartment of the skin. These mice show a rapid growth of hair follicles that is then followed by a disappearance of the epidermal stem cell compartment, progressive premature hair loss, and epithelial stem cell senescence. Treating the mice with rapamycin (4 mg/kg, i.p. for 18 days) prevented the accumulation of senescent epithelial stem cells, which in turn prevented long-term Wnt1-induced hair loss in the K5rtTA/tet-Wnt mice. Hinojosa et al. [110] studied the effect of rapamycin (4.7, 14, and 42 ppm) on p21 expression in the lungs of old (22 months) UM-HET3 mice. Cell senescence was reduced by all three doses of rapamycin. Herranz et al. [106] studied the effect of rapamycin on the paracrine effects of senescent cells on the tumorigenic potential of cancer cells using a mouse model of oncogene-induced senescence, *Nras*^*G12V*^ mice. Nras^G12V^ expression induced senescence in liver, and the SASPs produced by the senescent cells trigger an immune response in these mice. Rapamycin treatment (1 mg/kg by gavage once every 3 days) reduced SASP production in the *Nras*^*G12V*^ mice. In studying the effect of rapamycin on various aspects of cardiac function in old mice, Lesniewski et al. [111] found that rapamycin treatment (14 ppm) for 6 to 8 weeks reversed the age-related increase in the senescence marker, p19, in the aorta of old (~ 30 months) male B6D2F1 mice. Chen et al. [108] studied the role of cell senescence in pulmonary fibrosis using mice treated with bleomycin (intratracheally). Rapamycin (5 mg/kg, i.e., every other day a week after bleomycin treatment) was found to suppress the expression of senescence markers that were induced by bleomycin treatment. In addition, the rapamycin-treated mice showed reduced collagen deposition and pathological lesions in the lungs of the bleomycin-treated mice. A study with human subject > 40 years of age by Chung et al. [112] showed that the topical rapamycin reduced cellular senescence (p16^INK4A^ expression) in the skin that was accompanied by an improvement in the clinical appearance of the skin.

## Summary

In the 10 years since the initial report that rapamycin increased the lifespan of mice, there has been an explosion in the number of reports studying the effect of rapamycin on various parameters related to aging in mice. These studies have focused on determining the overall impact of rapamycin on aging processes and identifying potential mechanisms responsible for rapamycin’s pro-longevity effect. As a result of the data generated, it is now clear that there is a consensus in many areas as to the impact of rapamycin on mice, and the research reports in these areas have been described in this review. The first and most important outcome of these studies has been the demonstration that rapamycin has a robust effect on the lifespan of mice. Thirty studies have been conducted since 2009 showing rapamycin increases the lifespan of various strains and genetic models of mice (Tables 1 and 2). Currently, there are only three genetic mouse models where rapamycin has been reported to decrease the lifespan of the mice, i.e., ~ 90% of the reports that have studied the effect of rapamycin on lifespan in mice have shown a significant increase. One of the unexpected results from the lifespan studies is that rapamycin is effective over a broad range of doses in mice; doses much higher (threefold to 10-fold) than that initially shown to increase lifespan (14 ppm), i.e., rapamycin toxicity does not appear to be problematic in mice. Unfortunately, most of the reports studying the effect of rapamycin in mice have used only the lower, 14 ppm, dose of rapamycin. Based on the lifespan data, one might expect greater differences in the parameters that have been studied when higher doses of rapamycin are used.

When rapamycin was shown to increase the lifespan of mice, one of the first questions raised was whether this increase was due to rapamycin’s effect on aging. One of the ways to approach this question is to determine if rapamycin has a broad effect on processes directly related to aging, e.g., incidence of diseases. In other words, does rapamycin reduce/delay age-related diseases as well as increase lifespan? As shown in this review, the large amount of data in mice shows that rapamycin has a major impact on cancer, cardiac diseases and function, and normal brain aging including brain vascular aging and neurodegenerative-like processes in neurodegenerative diseases. In addition, rapamycin attenuates cell senescence in a broad range of cell types. Thus, rapamycin appears to have an anti-aging impact on a large number of disease-related processes in mice. Consequently, rapamycin is the first drug shown to have anti-aging actions in a mammal.

One of the intriguing aspects of rapamycin’s actions is that it is effective when given in later life. In the initial study by Harrison et al. [6], it was shown that rapamycin increased lifespan when administered to 19-month-old mice. Interestingly, the current data show that rapamycin is as effective increasing lifespan late in life as when it is given earlier in life. Additional studies not only show that rapamycin can be effective later in life but also that the effect of rapamycin can persist after treatment [113], i.e., mice do not have to be continuously treated with rapamycin for it to have an effect. For example, Bitto et al. [20] showed that treating 20-month-old mice with a high dose or rapamycin for only 3 months resulted in a dramatic increase in lifespan. In addition, they found that changes in the microbiome induced by rapamycin in these mice persisted after rapamycin treatment was discontinued. Several other investigators have shown that late life rapamycin treatment can reverse some age-related deficits in several physiological functions. For example, 10 to 12 weeks of rapamycin treatment reversed the age-related decline in cardiac function in 24- to 25-month-old mice [58, 59], and the improvement in cardiac function persisted for 2 months after rapamycin treatment was discontinued [113]. Lesniewski et al. [111] showed that 6 to 8 weeks of rapamycin reversed the age-related vascular dysfunction in 30-month-old mice. The age-related decline in cognition was also reversed when old (~ 20 months) mice were treated with rapamycin for 6 to 16 weeks [77, 79]. From a translational stand-point, these data are exciting because it suggests that rapamycin not only can reverse many of the adverse aspects of aging late in life but also need not be continuously given; its effect might persist well after it is discontinued. This also has recently been observed in elderly human subjects. In a study where 264 elderly subjects were given the rapalog, RAD001 for 6 weeks, Mannick et al. [97] found that the antibody titers to influenza virus vaccine were significantly higher in the rapamycin-treated subjects a year after giving rapamycin and the infection rates over the year were significantly reduced.

## Conclusion—where do we go from here?

The current mouse data conclusively demonstrate that rapamycin is effective in preventing/reversing a broad range of age-related conditions, including lifespan with minimal adverse effects or toxicity. However, there is always a concern as to how well discoveries in mice translate to humans. Currently, there are ongoing studies on the effect of rapamycin on companion dogs (by Matt Kaeberlein and Daniel Promislow at the University of Washington) and the non-human primate, the common marmoset (by Adam Salmon at the University of Texas Health Science Center at San Antonio). Salmon’s group recently reported that 9 months of rapamycin treatment had minor effects on clinical laboratory markers (e.g., plasma levels of glucose, cholesterol, triglycerides, and C-reactive protein did not change significantly) in middle-aged male or female marmosets [114]. Therefore, we are at a point when the aging community should began seriously considering clinical trials to test the anti-aging properties of rapamycin in humans as has been argued by Kaeberlein and Galvan [115] and Blagosklonny [87]. A major advantage of taking rapamycin to the clinic is the large amount of data gathered over the past two decades on the effect of rapamycin and its rapalogs on humans. The side effects of rapamycin in humans are well established, e.g., ulcers of mouth and lips, hyperglycemia/diabetes, hyperlipidemia, and hypercholesterolemia [116–118, 123]. In addition, the toxicity profile of rapamycin is relatively low in humans [119]. In addition, rapamycin is approved by the FDA for use in humans for transplantation and pancreatic cancer. Monica Mita (Cedars Sinai in Los Angeles), who has studied extensively the use of rapamycin and rapalogs in cancer therapy, has concluded, “we all have seen patients benefiting from the treatment with rapalogs and doing remarkably well for prolonged time with almost no change in the quality of life” [120]. In the past 2 years, two groups have specifically tested the feasibility of giving rapamycin to older subjects. As noted above, Mannick et al. [97] found that the rapalog, RAD001, was safe when given to subjects ≥ 65 years of age for 6 weeks; the RAD001-treated group actually showed improved response to influenza vaccination and reduced infections. In a pilot study with subjects 70 to 95 years of age who were otherwise healthy, Kraig et al. [121] found that 8 weeks of rapamycin was safely tolerated, e.g., the subjects showed no changes in cognitive or physical performance and in self-perceived health status. Importantly, they found that rapamycin had no significant effect on glucose tolerance or plasma triglyceride levels. Transplant patients receiving immunosuppressant regimes containing rapamycin have been reported to become diabetogenic [122] and have increased blood triglyceride levels [47]. However, as Dumas and Lamming (2019) [123] have pointed out, when taking rapamycin to treat human conditions related to aging, the side effects and the risk-benefit trade-off need to be considered. For example, the side effects are viewed as acceptable in treating cancer [124, 125] and would be acceptable in treating Alzheimer’s disease because there is currently no effective treatment.

So where do we go from here? We believe one of the first areas that should be seriously considered is taking rapamycin (or its rapalogs) to the clinic as a potential treatment of Alzheimer’s disease, as has been proposed by Kaeberlein and Galvan [115]. Currently, there is no treatment for Alzheimer’s disease. As is evident from Table 5, there is a large amount of data over the past decade showing that rapamycin prevents loss of cognition as well as Aβ and tau pathology seen in mouse models of Alzheimer’s disease, e.g., 10 studies using 7 different mouse models. The Moriss water maze, which was used to measure cognition in these studies, is comparable with clinically detectable, clinically relevant cognitive deficits in humans with Alzheimer’s disease. Rapamycin also has a beneficial effect on other neurodegenerative diseases as well as a wide variety of conditions that impact the central nervous system. Because age is the major risk factor in Alzheimer’s disease and rapamycin delays aging as shown by its effect on lifespan as well as many age-related diseases and physiological conditions and because rapamycin has a major impact on the central nervous system, we believe that rapamycin is a prime candidate for testing as a treatment for Alzheimer’s disease in humans. However, before taking rapamycin to clinical trials, it is important that additional pre-clinical data be gathered to more clearly define the effect of rapamycin on Alzheimer’s disease. Therefore, we suggest the studies described below that would generate additional data important for taking rapamycin to a clinical trial.

- *Determine whether rapamycin’s effect is sex dependent in transgenic mouse models of Alzheimer’s disease*: Almost all of the current studies did not identify the sex of the mice used, suggesting they used both sexes. Currently, there is no study specifically comparing the effect of rapamycin on male and female mice for any transgenic AD mouse model. As described above, rapamycin has a sex effect on longevity; the lifespan of female mice is increased more than male mice. In addition, it is well documented that gender plays an important role in Alzheimer’s disease: women are at a greater risk [126]. Therefore, defining how rapamycin effects the neuro-pathology and loss of cognition in male and female mice is important to know before taking rapamycin to human patients,
- *Define timing of rapamycin administration on cognition and pathology in transgenic AD mouse models*: Eight of the ten studies showing that rapamycin treatment attenuated Alzheimer’s disease were conducted early in the life of the mice; mice were treated with rapamycin before a significant cognitive deficit or amyloid burden occurred. While these studies show that rapamycin can prevent the development and progression of Alzheimer’s disease in mice, there is only limited information on whether rapamycin can reverse Alzheimer’s disease. Galvan’s group [127] studied the effect of rapamycin treatment (16 weeks) on 7-month-old mice hAPP (J20), which shown Aβ toxicity and loss of cognition. Rapamycin (14 ppm) restored brain vascular integrity and cerebral blood flow, decreased amyloid burden, and improved cognitive function. Jiang et al. [128] studied 5-month-old APP/PS1 mice, an age where these mice show the development of amyloid plaques and early cognitive deficits [129]. When these mice were treated with the rapalog, temsirolimus (20 mg/kg, i.p., every other day) for 2 months, Aβ clearance was enhanced and cognition improved. Thus, these two studies indicate that rapamycin (or its rapalog) can reverse the early effects of Alzheimer’s disease in mice. However, as Carosi et al. [130] have noted, these mice would be similar to state of Alzheimer’s disease in humans that might not be detectable. The one study that has evaluated the effect of rapamycin on Alzheimer’s pathology in old mice was conducted by Majumder et al. [65]. They studied the effect of treating 16-month-old 3xTg-AD mice with rapamycin (14 ppm) for 3 months. They found that rapamycin had no effect of the levels of Aβ or tau pathology or cognition in the 18-month-old 3xTg-AD, indicating that rapamycin was not able to reverse later stages of Alzheimer’s disease. Because rapamycin treatment late in life can increase lifespan [6] and reverse the age-related decline in cardiac function [58, 59, 113], vascular dysfunction [111], and cognition [77, 79] in mice, it would be important to repeat these studies using different mouse models of Alzheimer’s disease.
- *Effect of higher levels of rapamycin on Alzheimer’s disease*. All of the previous studies on Alzheimer’s disease and cognition used either 14 ppm or a similar, relatively low dose of rapamycin. It is now apparent that mice not only tolerate higher doses of rapamycin but that higher doses of rapamycin result in improved lifespan [19, 20, 24, 35, 131]. Therefore, it is possible that the inability of Majumder et al. [65] to see an effect of rapamycin on Aβ and tau pathology and cognition in the 18-month-old 3xTg-AD arose because the dose of rapamycin was too low. Thus, it is important to establish the optimum dose of rapamycin to treat Alzheimer’s disease.
- *Study the effect of rapamycin on other animal models*: Because many interventions that work in mice do not translate to humans, it is important to determine if the positive effects of rapamycin are seen in other animal models. For example, it would be relatively straight forward to studying the effect of various levels of rapamycin at early and late stages of Alzheimer’s disease using the transgenic AD rat models [132, 133]. As Carter et al. [134] have pointed out in a recent review, rats and mice differ in many parameters including pathology and performance on cognitive tests and are often more comparable with humans than mice. For example, rats have six tau isoforms, as do humans, while the mouse expresses only 4 isoforms [135]. In addition, the transgenic AD rat models currently available show Aβ and tau pathology and reduced cognition later in life than mouse models, which show pathology and cognitive deficits within 3 or 5 months of age. In contrast, sporadic Alzheimer’s disease occurs late in life. Therefore, the rat models of Alzheimer’s disease would be excellent models to establish whether the protective effects of rapamycin are consistent in rats and mice. In addition to studying the effect of rapamycin on rodents, it would be important to study other animal models. As described above, research is underway studying the effect of rapamycin on companion dogs and marmosets. Although these models do not get Alzheimer’s disease, marmosets at old age naturally develop amyloid deposits [136, 137].
